# Neurodegenerative Diseases: Multifactorial Conformational Diseases and Their Therapeutic Interventions

**DOI:** 10.1155/2013/563481

**Published:** 2012-12-30

**Authors:** Saba Sheikh, Safia, Ejazul Haque, Snober S. Mir

**Affiliations:** Department of Biotechnology, Integral University, Kursi Road, Lucknow, Ultar Pradesh 226026, India

## Abstract

Neurodegenerative diseases are multifactorial debilitating disorders of the nervous system that affect approximately 30 millionindividuals worldwide. Neurodegenerative diseases such as Alzheimer's, Parkinson's, Huntington's, and amyotrophic lateral sclerosis diseases are the consequence of misfolding and dysfunctional trafficking of proteins. Beside that, mitochondrial dysfunction, oxidative stress, and/or environmental factors strongly associated with age have also been implicated in causing neurodegeneration. After years of intensive research, considerable evidence has accumulated that demonstrates an important role of these factors in the etiology of common neurodegenerative diseases. Despite the extensive efforts that have attempted to define the molecular mechanisms underlying neurodegeneration, many aspects of these pathologies remain elusive. However, in order to explore the therapeutic interventions directed towards treatment of neurodegenerative diseases, neuroscientists are now fully exploiting the data obtained from studies of these basic mechanisms that have gone awry. The novelty of these mechanisms represents a challenge to the identification of viable drug targets and biomarkers for early diagnosis of the diseases. In this paper, we are reviewing various aspects associated with the disease and the recent trends that may have an application for the treatment of the neurodegenerative disorders.

## 1. Introduction 

**Figure 1:**
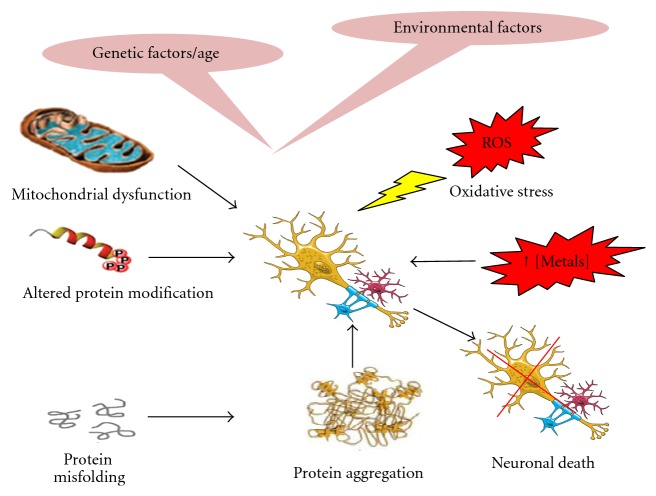
Different factors associated with neurodegenerative disease.

Neurodegenerative diseases such as Alzheimer's (AD), Parkinson's (PD), and Huntington's diseases (HD) and amyotrophic lateral sclerosis (ALS) make up a group of pathologies characterized by separate etiologies with distinct morphological and pathophysiological features. There is a huge body of evidence that suggest that these disorders arise by multifactorial conditions such as (a) abnormal protein dynamics with defective protein degradation and aggregation, (b) oxidative stress and free radical formation, (c) impaired bioenergetics and mitochondrial dysfunction, and (d) exposure to metal toxicity and pesticides (Figure 1). Although a lot of research has been carried out to understand the pathophysiology of these proteinopathies, still clarity in terms of viable drug targets is elusive. However, in order to explore applications directed toward developing recent emerging therapies for these diseases, neuroscientists have exploited the understanding of the basic etiology of these diseases. Although each disease has its own molecular mechanism and clinical manifestations, some general pathways might be recognized in different pathogenic cascades. They include protein misfolding and aggregation, oxidative stress and free radical formation, metal dyshomeostasis, mitochondrial dysfunction, and phosphorylation impairment, all occurring concurrently.

## 2. Consequences of Protein Folding and Misfolding

Proteins determine virtually every aspect of life by means of their highly diverse enzymatic and structural properties. At the same time, proteins are vulnerable macromolecules in the physiological environment of living cells. Cellular proteins must assume and maintain their native three-dimensional conformations in order to be biochemically and functionally active. Partial folding or misfolding makes protein functionally inactive, which may make the protein toxic to the cell. Truncated translational polypeptide products, misfolded intermediates, and unassembled subunits of oligomeric protein complexes frequently expose hydrophobic regions that have a tendency to form an aggregation or heap [1, 2]. Due to the hydrophilic nature of the cellular medium, hydrophobic surfaces from different misfolded proteins tend to interact with each other and to form cellular aggregates [3]. Protein misfolding followed by self-association and subsequent deposition of the aggregated proteins has been observed in the brain tissues of patients affected by these disorders. The biophysical behavior of these proteins, leading to their misfolding, aggregation, and deposition, has prompted scientists to group these kinds of neurological disorders under the common name of “conformational diseases” or proteinopathies.

Although protein biogenesis, for instance, is an error-prone process, to avoid alterations in protein homeostasis, cells possess high-fidelity protein quality control (PQC) pathways. There are two specific lines of defense to ensure the maintenance of proteostatic equilibrium inside the cell [4]. The first line of defense of the PQC systems consists of protein chaperones that bind to unfolded proteins, including newly synthesized proteins, and, by hydrolyzing ATP, actively aid in attaining mature protein conformation. A second line of defense of the PQC system clears proteins damaged beyond repair. This pathway includes E1-, E2-, and E3-ubiquitin ligases, which are recruited by the chaperones themselves and tetraubiquitinate irreversibly misfolded proteins, thus targeting them for proteolysis by the 26S proteasome. The PQC system also acts on mature properly folded but metastable proteins that have a tendency to revert to a nonnative state, particularly in conditions of proteotoxic stress such as in the presence of oxidizing agents or elevated temperature [1, 2]. A multitude of dedicated transcription factors also respond to proteotoxic stimuli by upregulating the transcription of genes that promote PQC. The PQC pathways are spatially compartmentalized according to the subcellular location of their misfolded substrates [5]. Molecular chaperones assist protein foldingand also facilitate degradation of the misfolded polypeptides by the ubiquitin-proteasome system [6]. Misfolding and aggregation are recognized as common molecular events for a large number of human diseases due to improper trafficking, premature degradation, or the appearance of toxic folds [7, 8]. Such diseases include CAG-repeat/polyglutamine (polyQ) expansion diseases including Huntington's disease (HD), Kennedy's disease, and spinocerebellar ataxias, and non-CAG diseases including Parkinson's disease, amyotrophic lateral sclerosis (ALS), prion disease, and Alzheimer's disease.

## 3. Role of Molecular Chaperones along with Autophagy in Maintaining Protein Homeostasis

In eukaryotic cells, protein homeostasis depends on molecular chaperones; these distinct functions are performed by two distinctly regulated chaperone networks [9]. Chaperones promote the folding of newly synthesized polypeptides, their translocation across membranes, and the refolding of stress-denatured substrates. Chaperones also play a key role in targeting misfolded proteins for degradation as well as preventing aggregation, they are generally classified according to their molecular masses as heat shock (HSPs) and small heat shock proteins (smHSPs) (e.g., hsp100, hsp90, hsp70, hsp60, and hsp40). Each family is comprised of multiple chaperone isoforms. Certain ATP-driven chaperones, such as Hsp70 and Hsp90 interact with cofactors, which directly influence their ATPase activity and direct them along different folding pathways [10, 11]. Chaperones, together with the autophagy and ubiquitin proteasome system (UPS), clear proteins which are abundantly expressed, yet there are evidences which suggest that limitations and malfunction of the clearance machinery are risk factors in diseases of protein conformation [12, 13]. Chaperones do not prevent formation of protein inclusions; instead of the formation of protein inclusion, chaperones are likely to inhibit the formation of toxic species by directing the misfolded species to nontoxic aggregates [14]. Chaperones have the ability to extract and refold proteins from aggregates beyond serving as a link between folding and degradation. The ring-shaped hexameric AAA-ATPases, specialized class of chaperones, can extract misfolded proteins from aggregates in an ATP-dependent manner [15]. The extracted protein can then be transferred to Hsp70 and Hsp40 chaperones for refolding or degradation [16–18]. Misfolded aggregated proteins can also be degraded by a separate autophagy pathway that involves their ultimate delivery to the lysosome [19]. Autophagy is a nonspecific bulk degradation pathway that was initially described for long-lived cytoplasmic proteins and damaged organelles [20]. This process is also a major degradation pathway for many aggregation-prone proteins associated with neurodegenerative disorders [21]. Knockdown of the autophagy genes (e.g., Atg5 and Atg7) leads to aggregation and neurodegeneration in certain mouse models [22, 23]. Conversely, upregulation of autophagy can play a protective role in quality control because it can promote the clearance of soluble small oligomeric aggregates, for instance, in HD models [24]. The multidimensional functioning and the capacity of molecular chaperones and degradative clearance machinery suggest that protein quality control is an efficient process; nevertheless the late onset of neurodegenerative diseases is indicative of the fact that these protein quality control mechanisms become overwhelmed with age and hence contribute to the disease conditions.

## 4. Mitochondrial Dysfunction, ROS, and Neurodegenerative Diseases

Small oligomeric aggregates and amyloid oligomers have been widely reported to permeabilize both cell and mitochondrial membranes. They are, therefore, responsible for calcium dysregulation, membrane depolarization, and impairment of mitochondrial functions, which have been identified as a further common feature of most neurodegenerative disorders. Neurodegenerative diseases are a consequence of genetic mutations and/or environment factors which are strongly associated with age [25, 26]. Mitochondrial dysfunction and oxidative stress play an important role for the development of the more common neurodegenerative disorders. Loss of mitochondrial function is associated with an increase in the generation of reactive oxygen intermediates and a number of human diseases [27]. Mitochondria use metabolic intermediates generated during the tricarboxylic acid (TCA) cycle to generate adenosine triphosphate (ATP) during oxidative phosphorylation. During the ETC (electron transport chain), electrons are occasionally captured by oxygen to produce superoxide anion radicals (O^−2^). Within the mitochondria, these superoxide radicals are converted to hydrogen peroxide by the action of manganese superoxide dismutase. Hydrogen peroxide in the mitochondria is broken down to water by the action of glutathione peroxidase or peroxiredoxins. The inhibition of ETC proteins can cause a subsequent increase in ROS resulting in decrease in the mitochondrial membrane potential, loss of ATP, and energy collapse and subsequent cell death [28, 29]. Beside the antioxidant enzymatic activities mentioned earlier, cells have nonenzymatic (GSH, vitamin E, vitamin C, and ubiquinone) scavengers to protect them against ROS. The result of an imbalance between ROS production and antioxidant action is called oxidative stress [30, 31]. Former estimates based on isolated highly energized mitochondria have suggested that as much as 2–4% of the oxygen consumed by mitochondria is liberated as superoxide or hydrogen peroxide [32], but recent studies as well as extrapolation to whole cells suggest that these early estimates are too high, and it has been estimated that mitochondria under normal physiological cellular conditions are intimately involved in the production of ROS through one-electron carriers in the respiratory chain and probably produce one to two orders lower amounts of reactive oxygen species (ROS). Neuronal tissue is particularly sensitive to oxidative stress, and imbalance in prooxidant versus antioxidant homeostasis in CNS results in the production of several potentially toxic ROS, including both the radical and nonradical species that participate in the initiation and/or propagation of radical chain reactions. In AD, PD, HD, and ALS, oxidative damage is found in every class of biological molecules within neurons, spanning from lipids to DNA and proteins [33]. However, the administration of one or few antioxidants is too simplistic, as demonstrated by the several clinical studies that have shown modest success with antioxidants in the treatment of neurodegeneration. Thus, dysfunctional mitochondria, alterations in mitochondrial dynamics, increased ROS, mtDNA damage, and the loss of energy production are important contributors to the pathophysiology associated with several neurodegenerative diseases including Alzheimer's disease (AD), Parkinson's disease (PD), and Huntington's disease (HD), and cancer. 

## 5. Several Environmental Factors Could Be Associated with the Induction of Neurodisorders

### 5.1. Metals and Neurodegeneration

Metals have an important role in neurodegeneration. While transition metals are essential in many biological reactions, alterations in their homeostasis result in increased free radical production, which is catalyzed by iron, copper, or other trace redox active metals. In all cases, metal-mediated oxidative stress is also linked to mitochondrial dysfunction. The increase of iron in the brain associated with several neurodegenerative diseases may lead to an increased production of free radicals *via *the Fenton reaction. The intracellular iron is usually tightly regulated, being bound by ferritin in an insoluble ferrihydrite core. The neurotoxin 6-hydroxydopamine (6-OHDA) releases iron from the ferritin core by reducing it to the ferrous form. In the presence of ferritin, both 6-OHDA and THB strongly stimulate lipid peroxidation, an effect abolished by the addition of the iron chelator deferoxamine. It suggests that ferritin iron release contributes to free-radical-induced cell damage in vivo. Iron accumulation could be an important contributor to the oxidative damage of AD [34] while an observation by some researcher suggests that dietary fat and a systemic defect in iron metabolism may act synergistically in the process of lipid peroxidation in PD [35]. Redox-active iron is associated with the senile plaques and neurofibrillary tangles, the pathological hallmark lesions of this disease. Iron associated with the lesion induces *in situ *oxidation and readily catalyzes an H_2_O_2_-dependent oxidation. Characterization of the iron binding site suggests that binding is dependent on the available histidine residues and on protein conformation. Iron can contribute to free radical damage by catalyzing the formation of the OH^•^, inducing secondary initiation of lipid peroxidation, and promoting the oxidation of proteins. The iron chelator, deferoxamine, can limit these oxidative reactions and it scavenges the peroxynitrite independent of iron chelation [36] because with deferoxamine the iron can be rebound to the lesions.

### 5.2. Pesticides Could Cause Neurologic Impairments in Elderly Persons

Pesticides encompass an array of compounds that are designed to kill insects and pests. Some work has been done by the researchers to investigate the hypothesis that the exposure to pesticides could be related to central nervous system disorders. Isabelle Baldi et al. studied that, in 1,507 French elderly people, lower cognitive performance was observed in subjects who had been occupationally exposed to pesticides in 1992–1998. In men, the relative risks of developing Parkinson's and Alzheimer's disease for occupational exposure assessed by a job exposure matrix were 5.63 (95% confidence interval: 1.47, 21.58) and 2.39 (95% confidence interval: 1.02, 5.63), respectively, after the confounding factors were taken into account. No association was found with having a primary job in agriculture or with environmental pesticide exposure nor was an association found in women. These results suggest the presence of neurologic impairments in elderly persons who were exposed occupationally to pesticides [37]. Several studies have shown positive association between pesticide exposure and the development of PD [37]. However, the specific compounds that lead to these varied effects are yet to be fully understood, and it is not very clear whether these pesticides share common molecular signatures that lead to neuronal toxicity. Therefore, investigators should continue to concentrate on identifying biomarkers for the improved estimation of pesticide exposure. The availability of such extensive molecular evidence raises the issue of cell-type specificity in neuronal disorders. Selective neuron degeneration has been shown to be a fundamental characteristic of each disease. The issue of cell-type specificity is, therefore, an open question for those studying the pathogenesis and treatment of these illnesses. New drug candidates with disease modifying potential are now in the pipeline and have reached testing in clinical trials. 

## 6. Therapies for Neurodegenerative Diseases

Stem cell therapy, gene transfer therapy, nanotechnology and medicinal chemistry based treatment, and multitarget directed ligands (MTDLs) have emerged as promising new therapies for neurodegenerative disorders.

### 6.1. Stem Cell Therapy and Neurodegenerative Diseases

Cell replacement therapy and gene transfer to the diseased or injured brain may act as potentially powerful new therapeutic strategies for human neurological diseases. Stem cells are capable of repairing injured nervous tissue by replacing damaged cells. They also offer neuroprotection or create an environment conducive to regeneration of endogenous cells [37]. The transplantation of stem cells may provide effective treatments due to the self-renewing and multipotent nature of these cells, including delivery of therapeutic factors to provide trophic support or missing gene products, mobilization of endogenous stem cells, and replacement of lost or dysfunctional cells. In Parkinson's disease, current therapies centere on the oral administration of L-dopa and dopamine receptor agonists and on deep-brain stimulation in the subthalamic nucleus [38]. Although the pharmacological treatment is effective for some symptoms, it has some limitations because its effectiveness decreases over time and side effects develop [39, 40]. Thus, an alternative approach for restoration of the damaged dopaminergic system is the transplantation of dopaminergic-synthesizing cells. Human stem cells may provide sources of cells for use in the treatment of PD [41]. One potential approach to prevent the death of existing neurons could be to transplant human stem cells engineered to express neuroprotective molecules such as the glial-cell-line-derived neurotrophic factor (GDNF) [42]. A recent strategy for treatment of HD has centered on cell therapy to protect vulnerable neuronal cell populations or to replace dysfunctional or dying cells [43]. Stem cell therapy aims to restore or preserve brain function by replacing and protecting striatal neurons. At this time, using stem cells for the delivery of trophic factors and the neuroprotection to prevent disease progression seems a more achievable clinical goal in HD than neuronal replacement. Most of the recent work in stem cell therapy has been conducted in animal models of HD. The study showed that cell replacement using grafts of fetal striatal neurons promoted functional recovery, and some evidence from clinical trials indicates that this could also occur in patients [44]. However, protocols and procedures developed from trials of fetal-derived cell transplantation in humans with HD lay the ground work to move stem cell therapy into the clinic. One of the first challenges to stem cell therapy in HD is to determine which source of stem cells is most efficacious, and many sources have been examined. In addition to human ESCs, stem cells derived from mesenchyme in adults have been investigated as a readily available source of stem cells in HD [45]. Although in Alzheimer's disease (AD) massive neuronal loss only occurs in very few brain structures, such as the hippocampal CA1 and CA2 regions, the entorhinal cortex, and the locus coeruleus, large parts of the brain are affected by pathological alterations and decreased neuronal metabolism [46, 47]. Current therapies, such as treatment with acetylcholinesterase inhibitors to enhance cholinergic function, provide only partial and temporary alleviation of symptoms [48]. The pathological changes seen in AD offer an extremely problematic situation for cell replacement. Because stem cells can be genetically modified to carry new genes and have high migratory capacity after brain transplantation, they could be used in place of fibroblasts for delivery of the nerve growth factor (NGF) to prevent degeneration of basal forebrain cholinergic neurons [49, 50]. The recent breakthroughs in stem cell research might nevertheless provide possibilities for neural implantation and cell replacement therapy for patients with amyotrophic lateral sclerosis (ALS). Kim et al. [51] showed that intrathecal injection with an optimized cell number could be a potential route for stem cell therapy in ALS patients. They suggested that, at this dose of 1 × 10^6^ cells/mL, the average number of motor neurons was significantly higher than others, and most injected hMSCs are distributed in the ventricular system and subarachnoid space. Additionally, the studies suggested that successful stem cell therapy for ALS likely would require that the cells be combined with other drugs or treatments, such as antioxidants and/or trophic molecules. 

Many exciting studies are taking this direction; both in vitro and in vivo studies have shown generation of motor neurons from human ESCs and functional engraftment of these motor neurons after transplantation into the developing chick and adult rodent spinal cord with axonal outgrowth toward muscle [52]. Recently, a Phase I clinical trial confirmed that MSCs transplantation into the spinal cord of ALS patients is safe and that MSCs might have a clinical use for future ALS cell-based clinical trials [53].  Recent studies have indicated that it is possible to generate motor neurons in culture from stem cells that include ESCs and NSCs. Mouse ESC-derived motor neurons transplanted into motor neuron injured rat spinal cord survived and extended axons into ventral root, and human ESCs transplanted into cerebrospinal fluid of rats with motor neuron injury migrated into spinal cord and led to an improved motor function [54]. However, factors that control the differentiation, survival, and maturation of stem cells in the context of a host degenerative brain must be more thoroughly understood before stem cell therapy will prove to be a robust and safe strategy that can be transferred to the clinic.

### 6.2. Treatment Employing Nanotechnology

Nanotechnology has proven to have great potential for providing neurotherapeutic modalities to limit and reverse the neuropathology of AD and PD by supporting and promoting functional regeneration of damaged neurons, providing neuroprotection, and facilitating the delivery of neuroactives such as drugs, genes, and cells across the blood brain barrier (BBB). It may contribute significantly toward the development of nano-enabled drug delivery systems for the treatment of NDs, taking advantage of the nanoscale structures of neural cells. Several novel approaches, inspired by recent advances in nanotechnology, are already applicable to the treatment of AD and PD. Nanoscale classes of neuroactives will widen the scope of therapeutic action beyond merely modifying transmitter function to include stem cell and gene therapies that could offer a more selective mode of targeting. Various potential nanostructures are employed for the treatment of neurodegenerative disorders such as polymeric nanoparticles, nanocapsules, nanospheres, polymeric nanogels, nanosuspensions, carbon nanotubes, nanofibers, polymeric nanomicelles, and polymeric nanoliposomes. PBCA nanoparticles have been used to deliver drugs to CNS, and gold nanoparticles have been successfully employed to destroy amyloid plaques in Alzheimer's disease [55].

### 6.3. Gene Transfer Therapies

 Several approaches to in vivo gene therapy for neurodegenerative diseases are currently being pursued both in animal models and in early human clinical trials. Gene transfer and novel approaches to in vivo gene therapy for neurodegenerative disorders were focused on (PD), (HD), and (AD). Genes can be delivered to the central nervous system (CNS) through the use of viral and nonviral vectors. Many viral vectors have been developed for this purpose, each with advantages and disadvantages. In general, viral vectors are delivered directly into the CNS via a craniotomy and are locally infused into specific neuroanatomical locations. Nonviral vectors such as liposomes may provide a means for delivering genes without the need for a craniotomy. The choice of vector and its mechanism of delivery will depend on the specific disease and its neurophysiology and the mechanism underlying the gene therapy. In vivo gene transfer therapy holds great promise for the treatment of neurodegenerative disorders. Recent success in human safety-tolerability trials will likely lead to an increasing number of human efficacy trials. Given the multitude of potential neuroanatomical, neurophysiologic, and genetic targets for interventions in neurodegenerative disorders, the possibilities for gene therapy are extensive. These possibilities will likely be expanded further as the techniques for viral and nonviral gene transfer to the CNS are improved. Many challenges exist for performing large-scale efficacy trials involving potentially risky genetic and neurosurgical interventions, including blinding and ethical considerations [56].

### 6.4. Medicinal-Chemistry-Based Strategies in the Treatment of Neurodegenerative Diseases

Medicinal-chemistry-based strategies include analogs as well as prodrug and codrug approaches. While each of these strategies may be equally promising to increase GSH levels, the codrug approach consists in linking, via a covalent chemical linkage, two different pharmacophores with similar or different pharmacological activities in order to improve the physiochemical, biopharmaceutical, and drug delivery properties of therapeutic agents. The resulting codrug has to be stable at gastrointestinal level and transported to the target site of action where it provides the two parent drugs following hydrolysis [57]. The codrug approach has been used for the treatment of PD and AD joining antioxidant or chelating molecules with a therapeutic compound (anti-Parkinson's or anti-Alzheimer's drugs) [58]. In particular, codrugs containing antioxidant molecules such as GSH, N-acetyl cysteine, methionine, and cysteinyl derivatives have been synthesized in order to permit a targeted delivery of antioxidant directly to specific groups of neurons where cellular stress is associated with PD and AD. The dual advantages of these antioxidant molecules lies in the fact that the antioxidant portion, in addition to acting as a scavenger directly or indirectly of free radicals, can be used as a carrier. In fact, GSH and cysteinyl derivatives can be used as blood brain barrier (BBB) shuttles for delivery of anti-Parkinson's or anti-Alzheimer's drugs since the presence of GSH transporters at the BBB is well documented [59]. Multitarget directed ligand (MTDL) design and discovery emerge as a possible alternative strategy for treatment of neurodegenerative disorders. MTDL may be a better candidate because it is able to hit several targets at once. Such an approach has recently been applied by some research groups to the discovery of new drug candidates for the treatment of PD. Curcumin and other polyphenols would appear to be useful in AD not only because of their dual function as anti-inflammatory and antioxidant agents but also because they can structurally interfere with *a beta-*aggregation and metal dyshomeostasis. There are numerous dietary factors that have been reported to affect brain physiology in ways that could, in theory, modify brain aging and the pathogenesis of neurodegenerative disorders. These range from amino acids such as tryptophan [60] to caffeine and related stimulants [61] (to omega-3 fatty acids) [62]. Gene array analysis of expression levels of thousands of genes in brains of young and old rats that had been maintained on control or restricted diets revealed changes in gene expression in brain cells during aging and showed that DR (dietary restriction) can suppress many of those changes. Age-related changes in the expression of genes that encode proteins involved in innate immune responses, oxidative stress, and energy metabolism are counteracted by DR. This retardation of brain aging at the molecular level may underlie the preservation of brain function during aging in animals maintained on DR. A recently published study has reported that DR prevents neurodegenration in an experimental model via a sirtuin mediated pathway [63] which might have implications for neurodegenerative Parkinson's disease. 

## 7. Conclusions

In light of the recent achievements in the fields of AD, PD, HD, and ALS, neurodegenerative diseases appear to share several common multifactorial degenerative processes that contribute to neuronal death, leading to functional impairments. Because of these multifactorial aspects and complexity, gene expression analysis platforms have been extensively used to investigate altered pathways during degeneration and to identify potential biomarkers and drug targets. Although many therapeutic approaches have been tested, no effective cure for these neurodegenerative diseases has been identified. Therefore, high-throughput techniques like whole genome transcriptomics and microarray technology must be coupled with functional genomics and proteomics in an effort to identify specific and selective biomarkers and viable drug targets, thus allowing the successful discovery of disease-modifying therapeutic treatments.
